# An In Vivo Comparison of Trueness and Precision of Two Novel Methods for Improving Edentulous Full Arch Implant Scanning Accuracy: A Pilot Study

**DOI:** 10.3390/dj12110367

**Published:** 2024-11-18

**Authors:** Adam Brian Nulty

**Affiliations:** 1 International Digital Dental Academy, London W1G 7JT, UK; dr@adamnulty.co.uk; 2 School of Dentistry, University of Leeds, Leeds LS2 9JT, UK

**Keywords:** intraoral scanners, photogrammetry, edentulous arch, dental implants, Scan Ladder system, accuracy and precision, full-arch scanning

## Abstract

**Background:** This retrospective in vivo study evaluated the trueness and precision of two digital intraoral scanners—Dentsply Sirona Primescan and Medit i900—, both with and without two variants of the novel Scan Ladder aids, and compared their performance to a new intraoral photogrammetry scanner (Shining 3D Elite). **Methods:** Data from ten edentulous patients, previously collected during routine clinical treatment, were analyzed using a master STL generated from traditional impression casts as the reference. A custom positional change calculator and comprehensive statistical analysis were used to assess scanner accuracy. **Results:** The findings demonstrated that the use of the Scan Ladder significantly enhanced the overall accuracy of both intraoral scanners, showing no statistically significant differences when compared to the intraoral photogrammetry system. **Conclusions:** These results indicate that the Scan Ladder improves the performance of conventional intraoral scanners and suggests that the Shining 3D Elite intraoral photogrammetry scanner is a reliable alternative to extraoral photogrammetry for edentulous cases. Further research, with a larger and more diverse cohort, is warranted to validate and expand upon these findings.

## 1. Introduction

The introduction of digital intra-oral scanners in the 1980s marked a revolutionary shift in dental practice, particularly within implantology. These devices offered a more efficient alternative to traditional tray-based impression techniques, enhancing the predictability of procedures [1]. As technology advanced, intra-oral scanners became central in modern dental practices, enabling same-day dentistry, reducing reliance on conventional methods, and improving treatment outcomes [2,3,4]. In implantology, integrating technologies like cone-beam tomography and computer-assisted planning has facilitated complete virtual patient modeling, improving treatment planning and diagnostic accuracy [5,6,7].

Despite these advances, intra-oral scanners face challenges in edentulous cases, where achieving the precision necessary for long-span or full-arch implant prostheses requires extremely high accuracy (trueness and precision) to ensure a passive fit [8]. Traditional scanning methods often struggle to capture edentulous arches due to the lack of distinct anatomical landmarks, complicating the stitching of scan frames [9].

Since the early 1990s, stereophotogrammetry (SPG) has been used to improve the accuracy of digital impressions in edentulous implant cases [10]. Although SPG has proven effective in capturing intraoral implant positions, studies report mixed results on its precision [11,12,13,14,15,16,17]. Comparative research evaluating various digital techniques remains scarce, highlighting the need for more in vivo studies to address these inconsistencies.

This study aims to evaluate the accuracy (trueness and precision) of two digital intra-oral scanners, Primescan and i900, using novel scanning methodologies alongside the Shining 3D Elite intraoral photogrammetry scanner. Accuracy, defined by trueness and precision, refers to the scanners’ ability to consistently reproduce implant positions [18,19]. To overcome the challenges associated with edentulous topographies, this research introduces the “Scan Ladder”, a novel device designed to enhance scan accuracy by providing distinct three-dimensional reference points, improving the scanner’s ability to capture complex topographies and ensuring precise data stitching [20,21,22,23].

By incorporating these advancements, this study evaluates the trueness and precision of digital intraoral scanners in capturing full-arch edentulous implant scans. The comparison involves conventional methods, the Scan Ladder, and the Aoralscan Elite with IPG, measured against a master STL obtained from a high-precision lab scanner. This research explores whether these novel methods improve implant position accuracy and provide potential solutions for achieving optimal outcomes in implant-supported prostheses. Additionally, it investigates the influence of these innovations on the accuracy of digital impressions in clinical practice.

In the following sections, we detail the methods, present the results, and offer a comprehensive analysis of our findings. This study tests the null hypothesis of no significant differences in trueness and precision between the scanners, both with and without the Scan Ladder and Elite intraoral photogrammetry. The analysis determines the extent to which these tools improve the accuracy of digital impressions in clinical settings.

## 2. Materials and Methods

### 2.1. Patient Recruitment

This retrospective study analyzed data previously collected during routine dental treatment and diagnostic procedures at Digital Smile Studio, Harley Street, London. Ten patients, all of whom had undergone prior treatment with multiple (≥4) dental implants and Multi-Unit Abutments, were included based on existing clinical records. These records, which were fully anonymized before analysis, involved scans taken at various points of the treatment pathway, from implant placement and the initial impression of the preliminary prosthesis, and scans taken for the final prosthesis with two variants of the “Scan Ladder” system: the Indirect Scan Ladder (attached to a conventional scan body) and the Titanium Direct Scan Ladder (a reusable matte surface scan body directly connected to the abutment).

No new data collection or interventions were performed for this study. Instead, this study relied on a comprehensive retrospective analysis of anonymized patient data. All participants had previously undergone scans using three intraoral scanners—Dentsply Sirona Primescan, Medit i900, and the Shining 3D Elite Intraoral Photogrammetry Scanner (IPG). These scans, taken with and without the Scan Ladder variants, were compared against a master STL generated by a high-precision structured light laboratory scanner (Ineos X5, Densply Sirona, Bensheim, Germany) taken as part of the verification process in the construction of the final prosthesis.

The study followed international ethical standards and involved the secondary analysis of anonymized data. No personal identifying information was available to the researchers in analyzing the results.

### 2.2. Master STL Creation

A master model was created using a laboratory study cast of an edentulous full arch derived from pre-existing patient treatment records. In this case, a preoperative digital scan was performed using an intraoral scanner to capture the initial anatomical details of the edentulous arch. Based on these digital data, a custom tray was digitally designed and 3D-printed to precisely fit the patient’s unique arch anatomy. This process ensured that the final impression would capture all relevant soft and hard tissue landmarks with enhanced accuracy.

For the final impression, polyvinyl siloxane (PVS) material, a highly accurate elastomer, was used in conjunction with the custom tray. PVS was selected for its superior dimensional stability and ability to produce highly detailed impressions, which is particularly important when capturing the smooth and feature-poor surfaces of an edentulous arch. To further ensure accurate border capture, border molding was performed with green stick compound, allowing for an optimal impression of the soft tissue periphery.

Once the impression was taken, type IV dental stone was poured into the mold to create a highly accurate physical model of the edentulous arch. This positive model was then scanned with the Ineos X5, a high-precision structured light scanner accredited for accuracy within 2.1 microns (ISO 12836) [18]. The resulting Master STL file generated from the scan served as the reference standard for all comparative analyses within the study.

The aim of this study was to quantitatively assess the trueness (accuracy) and precision of intraoral scanners, including the Elite Intraoral Photogrammetry (IPG) system, by comparing their outputs to the Master STL. By comparing the digital impressions to this highly accurate reference, this research focused on evaluating the impact of the Scan Ladder system in improving digital impression accuracy for edentulous arch scenarios, where precise implant positioning and passive fit are critical for the success of implant-supported prostheses.

### 2.3. Scanning Procedure

The retrospective analysis included intraoral scans performed under standardized conditions with three scanners—Primescan, i900, and the Shining 3D Elite IPG—capturing implant positions in ten edentulous arch cases. These scans were taken in three configurations: (1) without scanning aids, (2) with the indirect Scan Ladder, and (3) with the direct Scan Ladder. Lab-based scans generated by the Ineos X5 were used to create the Master STL files as part of the treatment verification process, which were used to evaluate the accuracy and precision of the intraoral scans. By comparing the intraoral scans to this high-precision benchmark, this study quantitatively assessed the effectiveness of the Scan Ladder in enhancing digital impression accuracy for edentulous arches.

**Figure 1:**
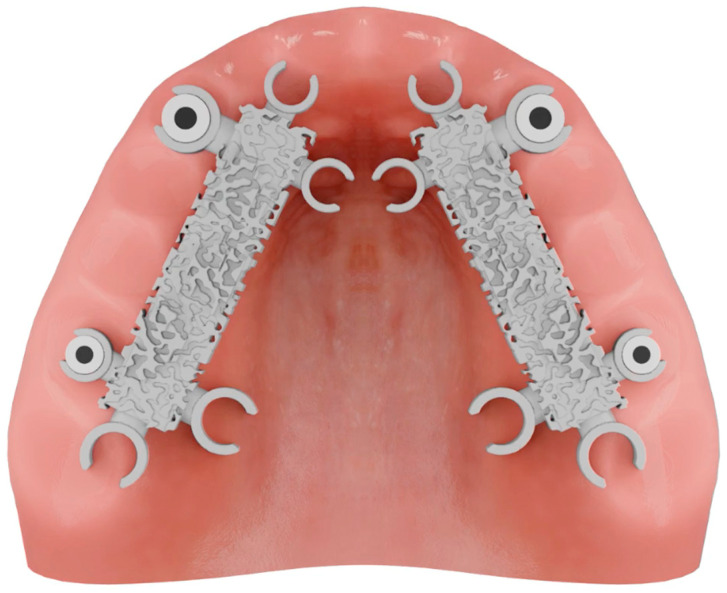
The Scan Ladder indirect variant.

**Figure 2:**
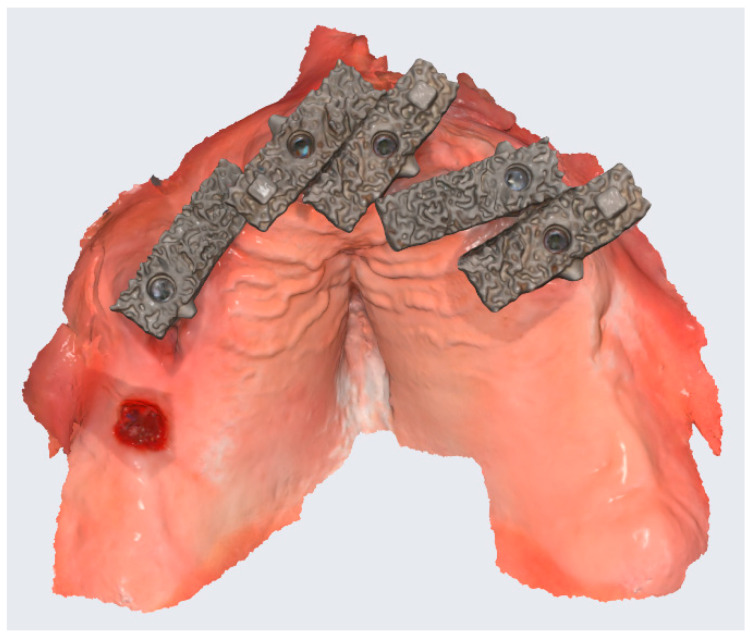
The Scan Ladder titanium direct variant OBJ scan as scanned on a Medit i900.

To improve the accuracy of the scans in edentulous arches, this study introduces a novel method. This method involved the use of a “Scan Ladder”, as depicted in Figure 1, and a second variant of the “Scan Ladder” that connects directly to a multi-unit abutment and acts as a scan body in itself, as depicted in Figure 2.

This study introduces two variants of the Scan Ladder: the indirect variant, which functions as a scanning aid by attaching to conventional scan bodies, and the direct variant, which directly connects to a multi-unit abutment and serves as a scan body (Figure 1 and Figure 2). The titanium direct variant further enhances precision with its reusable matt surface, allowing more accurate referencing within CAD software such as Exocad 3.2. These innovations improve the capture and digital modeling of edentulous arches for implant-supported prostheses.

**Figure 3:**
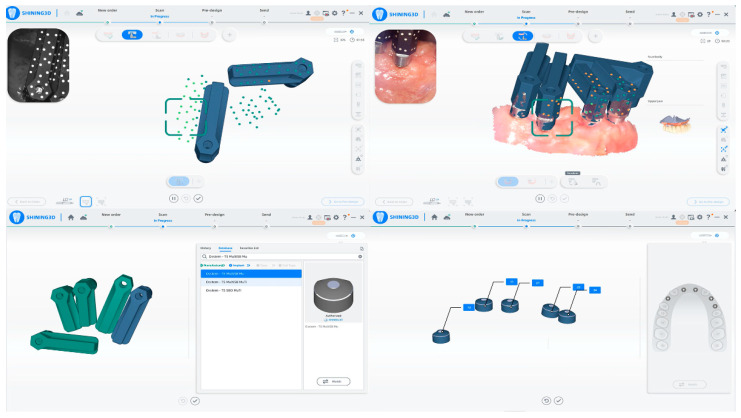
Elite IPG utilizing a coded pattern to calculate each scan body position in relation to the others.

In addition to the Scan Ladder, this study integrates the Shining 3D Aoralscan Elite, an intraoral scanner featuring intraoral photogrammetry technology (IPG). IPG enhances accuracy by combining photogrammetry with intraoral scanning capabilities, making it particularly useful in All-on-X treatments. Using coded patterns on scan bodies as accuracy control points (Figure 3, upper left panel), IPG ensures consistent global accuracy while minimizing the need for labor-intensive multi-angle scanning, streamlining the workflow.

The Aoralscan Elite overcomes the limitations of conventional photogrammetry systems, which cannot simultaneously capture intraoral gingival scans, by merging data sets using artificial intelligence (Figure 3, upper right panel). This AI-driven system automates data processing, exporting virtual scan body data sets compatible with specific implant systems (Figure 3, lower left panel and lower right panel). The integration of IPG with real-time dynamic tracking ensures precise calculation of implant positioning, enhancing patient comfort and diagnostic accuracy through the delivery of high-resolution data.

### 2.4. Design of the Study

#### 2.4.1. Overview

This retrospective in vivo study evaluated the accuracy (trueness and precision) of two advanced intraoral scanners—Primescan (Dentsply Sirona, York, PA, USA) and i900 (Medit, Seongbuk-gu, Seoul, Korea)—as well as the Elite Intraoral Photogrammetry Scanner (Shining 3D Tech Co., Hangzhou, China). The trueness and precision of each scanner, both with and without the Scan Ladder variants, were compared against a Master STL for each implant position.

The Master STL was generated with each scanner and validated using the Ineos X5 Lab Scanner, which is ISO 12836 accredited with an accuracy of 2.1 microns [24]. A sample size of 10 was determined, providing statistically significant results with a 95% confidence level and a 5% margin of error, confirmed by multiple studies [18,25,26].

#### 2.4.2. Scanners Used in This Study

##### Scanner Specifications

This study employed a range of scanners with distinct technologies. Their key features are summarized below:

- Primescan: Utilizes structured light technology with confocal microscopy and a Smart Pixel sensor. It supports STL export and indirect PLY/OBJ color export.
- i900: Uses structured light technology with Active Speed 3D Video™, improving the speed and accuracy of data capture. It supports both STL and PLY/OBJ export.
- Elite: A novel intraoral photogrammetry scanner designed for full-arch implant scans with high precision, supporting STL and PLY/OBJ export.

These scanners were tested under identical conditions, allowing for a comprehensive and direct comparison of their performance in edentulous cases.

The scanners used in the present in vitro study are summarized in Table 1.

#### 2.4.3. Patient and Implant Details

The ten patients selected for this study had multiple dental implants fitted with multi-unit abutments from various brands, all utilizing IPD multi-unit abutments (Implant Prothesis Dental 2004 SL, Italy), featuring a Nobel-compatible universal design. This uniformity was essential for ensuring consistency in digital capture across participants.

Implant scan bodies compatible with the multi-unit abutments were placed on each implant. These scan bodies were critical for capturing accurate digital representations of the implant positions, ensuring that the spatial orientation and position of each implant were effectively reflected in the scans.

#### 2.4.4. Scanning Methodology

Each patient underwent a series of scanning procedures to capture implant positions as part of their treatment plan for full-arch implant bridge provision. Impressions of the implant positions were taken using both indirect and direct Scan Ladder systems alongside traditional scan bodies. Initially, the Primescan and i900 scanners captured the positions of the implant scan bodies attached to the IPD multi-unit abutments. Following these scans, the indirect Scan Ladder was used for each scanner, followed by the direct Scan Ladder. Lastly, the Elite Intraoral Photogrammetry Scanner captured the abutment positions through its novel abutment capture system as part of the final prosthetic design.

These scans were compared against a Master STL generated by the Ineos X5 structured light lab scanner, accredited for accuracy within 2.1 microns [24]. This master scan, taken as a verification impression in the treatment, served as the gold standard for comparison in the retrospective study, allowing the researchers to evaluate the added value of the Scan Ladder and Elite Scanner in improving accuracy. To ensure statistical robustness, each patient underwent five scans per type (without aids and with indirect and direct Scan Ladder), resulting in 10 results per scan configuration. This sample size was calculated to provide a 95% confidence level and a 5% margin of error, ensuring reliable data for statistical analysis [25,26,27]. This approach facilitated a detailed assessment of the potential improvements offered by the Scan Ladder and the Elite scanner.

#### 2.4.5. Operator and Scanning Technique

A single experienced operator conducted all scans to maintain consistency. The scanning protocol followed each manufacturer’s guidelines. Scanning started from the upper leftmost molar and moved occlusally across the arch, pivoting to capture palatal and buccal surfaces. This consistent methodology ensured uniform mesh alignment, enabling accurate data comparison.

#### 2.4.6. Data Processing and Analysis

Following the scanning procedures, each scan body was meticulously registered within the Exocad 3.2 CAD software. The relative positions of these scan bodies were exported in STL format, adhering to each manufacturer’s specific conversion recommendations. This standardized method ensured that the data retained its integrity and accuracy throughout the processing phase.

Once exported, the STL files were imported back into Exocad for alignment, ensuring the best fit with the existing soft tissue models. This step was critical to accurately juxtapose the digital impressions with the real anatomical features of the edentulous arches, thereby enhancing the precision of the overlays. This process resulted in a set of implant position STLs for each implant and for each type of scan, with the common alignment being the soft tissue, enabling direct comparison of the virtual implant STLs for each implant. The alignment and registration process was systematically repeated for each intraoral scan conducted in this study. To facilitate a structured analysis, the multi-unit STLs were categorized and named according to the scan type and individual implant positions (one through ten) and annotated to indicate whether they represented pre-planned or post-placed positions. This rigorous classification helped in creating an organized repository of STL files, distinctly categorized by their 3D XYZ coordinates, enabling a streamlined comparison.

**Figure 4:**
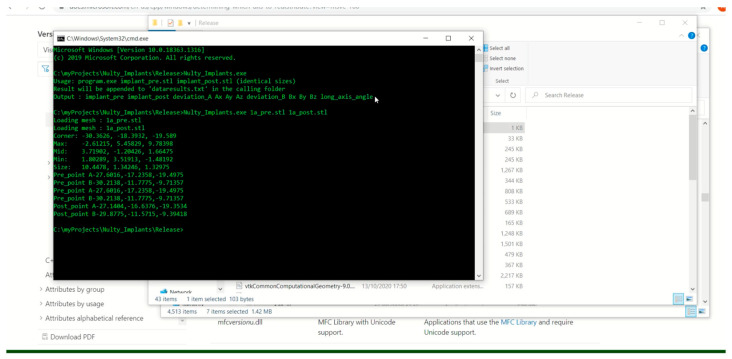
The positional change calculator.

**Figure 5:**
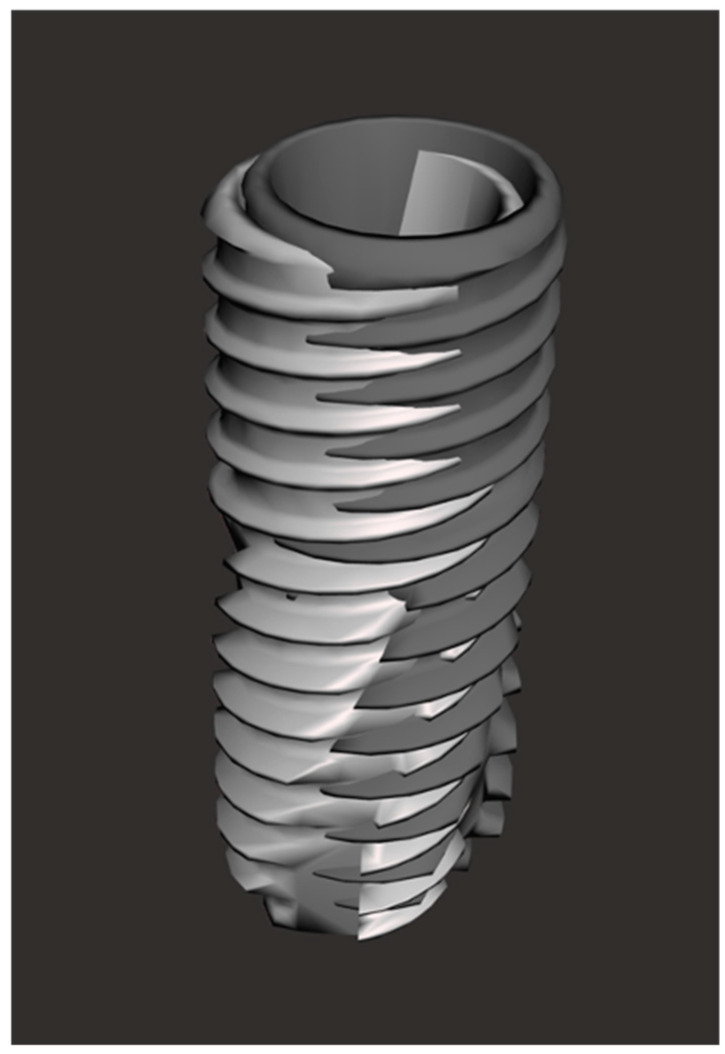
The positional change calculator uses a pair of identical virtual implant STLs to compare a point-for-point analysis of movement change.

For the quantitative analysis of these data, a custom-developed C++ program was employed, as shown in Figure 4. This program calculated the XYZ positional changes of each STL, as shown in Figure 5, focusing specifically on the deviations observed in the center of the coronal aspect of each multi-unit abutment across different scan types. This analysis was critical in quantitatively assessing the trueness and precision of each scanner and scanning aid used, particularly the Scan Ladder system, in real-world clinical settings, providing a clear picture of their impact on the accuracy of dental implant placements [28].

##### Evaluating Trueness

Trueness was assessed by measuring the deviation between the coronal positions of the virtual multi-unit abutments in each scan and the Master STL. These deviations were recorded for each implant position, reflecting how accurately each scanner replicated the known implant locations. The numerical deviations were compiled into a comprehensive table to facilitate the organized analysis of the trueness of each scanning method.

##### Evaluating Precision

Precision was quantified by evaluating the consistency of repeated scans using the same intraoral scanner. All pairwise comparisons between the scans were made to assess superimposition consistency. A one-way analysis of variance (ANOVA) was conducted to compare mean differences across scan groups, with post hoc multiple comparisons using Tukey’s HSD test (significance level of 0.05). Bartlett’s test was used to evaluate variance homogeneity across groups. These analyses provided clear measurements of the scanner’s consistency over multiple uses.

##### Statistical Analysis

Statistical analysis was performed using SPSS 29 software (IBM, SPSS Inc., Chicago, IL, USA) [29]. Since each scan session captured the positions of ten separate implants from ten participants, data were grouped and analyzed for each participant and scanning method. This granular approach enabled a thorough examination of any scanner-specific variations and potential anomalies, including those between the different scanning methods (Scan Ladder variants and the Elite IPG).

One-way ANOVA tests were employed to identify significant differences in trueness and precision across the different scanners and configurations, and post hoc tests were performed to explore specific intergroup variations further. This detailed segmentation allowed for isolating the factors that influenced scan accuracy, particularly in fully edentulous cases. The rigorous statistical methods strengthened the study’s conclusions, providing a solid foundation for evaluating new technologies and methods in dental implantology.

## 3. Results

**Figure 6:**
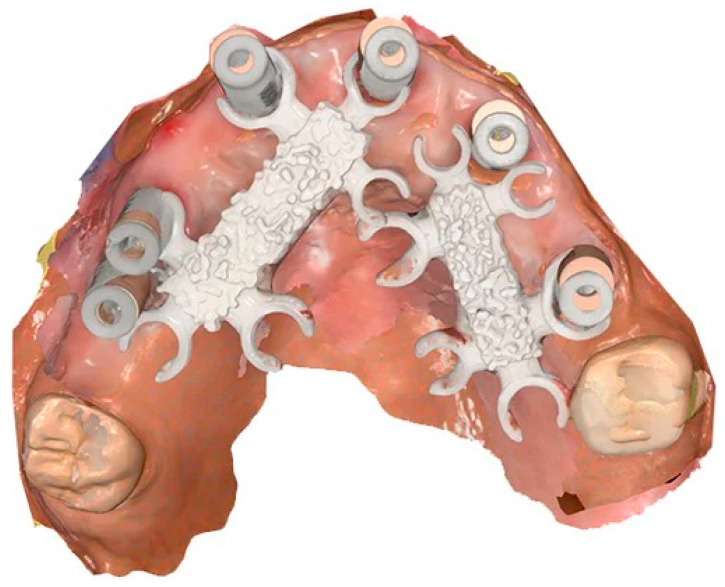
A scan overlay of the indirect Scan Ladder scan with a normal scan using the Medit i900.

The results demonstrated a marked improvement in accuracy with the use of the Scan Ladder, with deviations comparable to the Elite Intraoral Photogrammetry scanner. Traditional methods without scanning aids produced significantly higher deviations, highlighting the challenges of accurately capturing edentulous arches. These results affirm that advanced technologies like the Scan Ladder and Elite scanner can offer significant clinical benefits, particularly in ensuring passive fits in full arch implant-supported prostheses. The scan overlay with and without the indirect Scan Ladder system can be seen in Figure 6 below.

**Figure 7:**
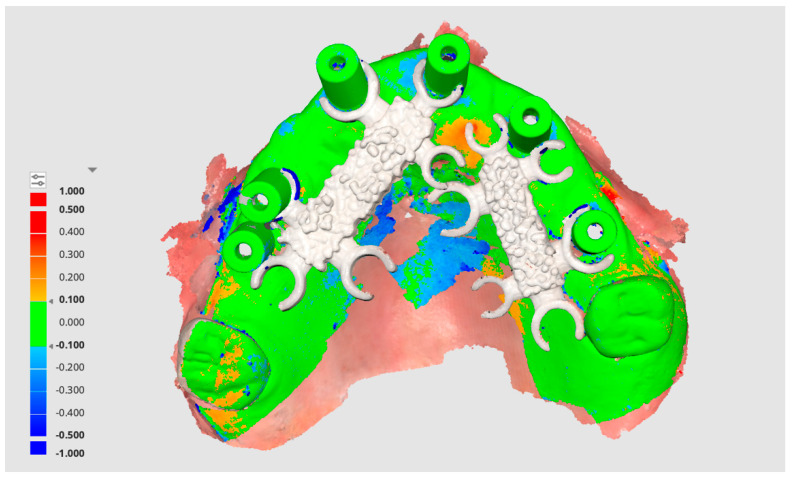
A scan overlay of the indirect Scan Ladder scan with the Master STL showing deviation between the two scans.

An overlay between an indirect Scan Ladder scan and the Master STL can be seen in Figure 7 below.

**Figure 8:**
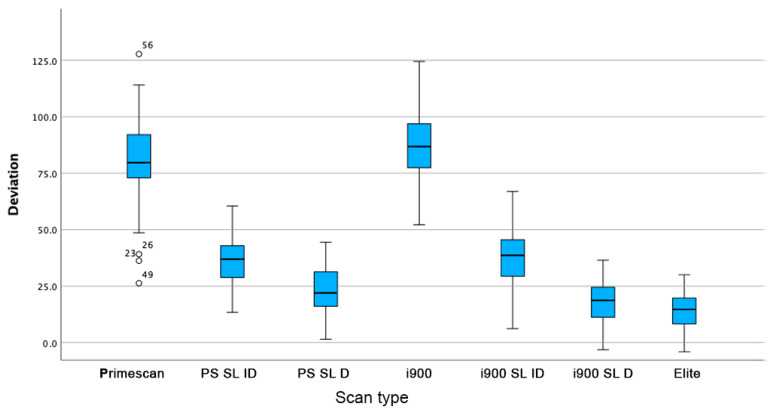
Box plot of deviation in μm for each data set for each scan type in the present study. Figure legend: Primescan: Primescan with standard scan body; PS SL ID: Primescan Scan Ladder indirect kit; PS SL D: Primescan Scan Ladder direct titanium kit; i900: i900 with standard scan body; i900 SL ID: i900 Scan Ladder indirect kit; i900 SL D: i900 Scan Ladder direct titanium kit; Elite: Elite IOP: intraoral photogrammetry.

Trueness and Precision results are summarized in Table 2 and Table 3 and in Figure 8.

Mean trueness: Represents the average deviation from the Master STL for each scanner, with lower values indicating greater accuracy. The Elite Intraoral Photogrammetry scanner exhibits the highest trueness. A box plot of the results is shown in Figure 8.

*p* value: Indicates statistical significance, with values of 1.000 suggesting no significant difference from other high-performing methods. The values of 0.052 for traditional scanning methods indicate less reliability without scanning aids.

In this study, the Elite Intraoral Photogrammetry scanner demonstrated the highest mean accuracy and precision across all tested devices. The performance of the Elite scanner was closely matched by the Primescan and Medit i900 when used with the direct Scan Ladder method, significantly improving their accuracy and bringing them close to the Elite’s results.

Quantitatively, the Elite scanner had the lowest mean deviation from the Master STL, followed closely by scanners using the direct Scan Ladder, with no statistically significant differences between the three data groups.

## 4. Discussion

### 4.1. Evaluation of Trueness and Precision in Digital Intra-Oral Scanners

This study rigorously assessed the trueness and precision of two leading intraoral scanners, the Primescan and i900, in the challenging context of full-arch scans for in vivo edentulous implant cases. Scanner accuracy plays a crucial role in ensuring the fit and long-term success of dental implants. Enhanced digital impression accuracy directly influences implant placement and prosthesis success, impacting the long-term prognosis of implant-supported restorations.

### 4.2. Contribution to Existing Scientific Literature

This research contributes valuable empirical data on digital intraoral scanner reliability and accuracy, particularly in edentulous cases. Prior studies highlight the potential of digital scanners to improve clinical outcomes, but their effectiveness varies depending on the anatomical challenges of the scanned arches and the specific technologies used [30]. By integrating the Scan Ladder and comparing traditional scanning methods with the Elite Intraoral Photogrammetry scanner, this study offers new insights into how different scanning technologies influence digital impression accuracy.

### 4.3. Importance of Deviation Metrics for Prosthesis Fit

Achieving a passive prosthesis fit is essential to minimize long-term implant complications. Studies suggest that deviations under 59 to 72 μm are required to ensure passive fit and mitigate biomechanical failure risks [31]. The use of novel methodologies with both the Primescan and i900, alongside the Elite Intraoral Photogrammetry scanner, significantly enhanced accuracy, with the following deviations:

- Elite: 18.7 ± 9.5 μm
- Primescan (direct Scan Ladder): 22.2 ± 10.6 μm
- Medit i900 (direct Scan Ladder): 22.7 ± 10.3 μm
- Traditional scanning methods showed significantly higher deviations:
- Primescan (traditional): 80.4 ± 18.3 μm
- Medit i900 (traditional): 86.9 ± 17.2 μm

The use of the titanium direct Scan Ladder method and the Elite scanner showed no statistically significant differences in mean deviations between them, underscoring the ability of these enhanced techniques to meet or exceed the standards necessary for passive fit. These results demonstrate the transformative impact of advanced photogrammetry and digital scanning technologies in improving accuracy for implant-supported prostheses.

### 4.4. Enhancement in Accuracy with the Scan Ladder

The findings revealed a statistically significant improvement in accuracy when using the Scan Ladder, crucial for restoring full-arch implant cases. Figure 6 and Figure 7 highlight the improvement in digital impression accuracy, showing strong alignment between the Scan Ladder-enhanced scans and the Master STL. This advancement is vital for achieving accurate virtual modeling of implant cases, improving passivity and functionality.

### 4.5. Comparison with Peer-Reviewed Literature on Photogrammetry

This study’s results align with recent literature on photogrammetry, highlighting the evolving capabilities of digital scanning technologies in implant dentistry. Peer-reviewed studies report significant challenges in capturing accurate impressions of edentulous arches, primarily due to the lack of stable reference points and feature-poor ridges [32,33]. The Elite Intraoral Photogrammetry scanner, which achieved a deviation of only 18.7 μm, and the direct Scan Ladder scan groups, with mean deviations of 22.2 μm and 22.7 μm, outperformed traditional methods, consistent with findings from Cheng et al. [33], who demonstrated that photogrammetry can match or exceed conventional scanner accuracy.

### 4.6. Review of Related Literature

Previous research has highlighted the inherent limitations of intraoral scanners in capturing highly accurate impressions, particularly in fully digital workflows [34,35,36,37]. Despite advancements, scanners face challenges in cases involving edentulous arches, where traditional lab-based scanning methods are often preferred. This study introduces innovative solutions like the Scan Ladder and Elite Intraoral Photogrammetry scanner, demonstrating their promise in overcoming these challenges. The Elite scanner’s deviation of only 18.7 μm surpasses traditional methods, aligning trueness closer to the Master STL, a crucial factor for achieving accurate and functional dental prostheses.

The Scan Ladder, both in its direct and indirect variants, significantly enhanced the performance of standard intraoral scanners by providing structured support. This improvement in scan accuracy aligns with recent literature that underscores the value of photogrammetry and scanning aids in improving intraoral scanner precision.

### 4.7. Implications for Clinical Practice

This study, in combination with related research, underscores the importance of selecting the right technology, optimizing operator skills, and implementing effective clinical strategies to enhance digital intraoral scanner accuracy. The Scan Ladder and Elite Intraoral Photogrammetry system demonstrated substantial improvements in trueness and precision for full-arch edentulous implant cases. These advancements directly address persistent challenges in implant dentistry, particularly in achieving precise prosthesis fit.

Accurate fits are crucial for avoiding complications such as implant misalignment, undue stress, or prosthetic failure. Incorporating tools like the Scan Ladder and Elite Intraoral Photogrammetry scanner into routine clinical workflows can significantly enhance treatment predictability and clinical outcomes in complex rehabilitations.

### 4.8. Challenges and Limitations of the Present Study

While this study demonstrates the potential of advanced scanning technologies like the Scan Ladder and Elite Intraoral Photogrammetry, several challenges and limitations remain:

- Algorithmic limitations: Intraoral scanners depend on distinctive landmarks for scan alignment. The smooth surfaces of edentulous arches pose challenges for accurate frame merging, potentially affecting model accuracy.
- Implant positioning and angulation: variations in implant depth and angulation complicate scanning, introducing potential inaccuracies.
- Operator expertise: operator skill was highly controlled in this study, but clinical results may vary based on operator proficiency.
- Sample size and diversity: a larger and more diverse patient cohort is necessary to validate these findings across different clinical scenarios.
- Arch characteristics: This study focused on maxillary arches. Future research should explore mandibular arches, which present additional scanning challenges due to less stable landmarks.

Addressing these limitations in future research will provide a deeper understanding of advanced intraoral scanning technologies like the Scan Ladder and Elite Intraoral Photogrammetry. Expanding to a prospective in vivo study with a larger patient cohort, exploring different clinical settings, and conducting comparative analyses will be essential for establishing the reliability and applicability of these technologies in clinical practice.

### 4.9. Results Interpretation

This study rigorously assessed the performance of two leading digital intraoral scanners, Primescan and i900, with a focus on their efficacy in scanning edentulous arches. A key aspect of this investigation was the introduction of the Scan Ladder and the novel Elite intraoral photogrammetry scanner to assess their role in enhancing digital impression accuracy.

Our findings revealed no significant difference in performance between Primescan and i900 when used with the Scan Ladder, highlighting that both scanners offer comparable accuracy for precise digital impressions in edentulous arch cases. The Scan Ladder’s integration substantially improved scan fidelity, bringing the results closer to the Master STL.

However, without the Scan Ladder, substantial deviations were observed, underlining the difficulty of capturing accurate impressions in edentulous arches without auxiliary tools—critical for precise implant dentistry. Additionally, the Elite Intraoral Photogrammetry scanner demonstrated excellent performance, achieving trueness with a mean deviation of just 18.7 μm.

### 4.10. Future Directions

Further research should expand on these findings by incorporating a broader range of scanners, more diverse patient populations, and direct comparisons with other digital impression techniques. Such studies will deepen our understanding of the current capabilities and limitations of intraoral scanning technologies. Exploring the Scan Ladder and Elite Intraoral Photogrammetry across varied clinical environments and larger sample sizes is critical for establishing their routine use in dental practice. Continuing to refine these technologies promises to enhance digital impression accuracy, ultimately benefiting both clinicians and patients by improving outcomes and procedural efficiency.

## 5. Conclusions

The findings of this pilot study refute both the primary and secondary null hypotheses, confirming that the Scan Ladder significantly improves the accuracy of digital impressions. Using the Scan Ladder enhanced the scanners’ performance, aligning them closely with the precision of a high-precision lab scanner and demonstrating minimal deviations overall.

The results highlight the Scan Ladder’s ability to reduce deviations and improve the trueness and precision of digital impressions, with the Primescan and i900 used with the direct Scan Ladder reaching 22.2 μm and 22.7 μm, respectively. This consistent improvement across different scanners signifies a meaningful advancement in digital dentistry, enabling more reliable full-arch passive impression scans when photogrammetry technology is not available. Notably, the Elite Intraoral Photogrammetry scanner demonstrated an exceptional level of accuracy, with a mean deviation of just 18.7 μm, underscoring the potential of advanced photogrammetry for producing highly precise digital impressions in edentulous arch cases.

The strong performance of the Elite scanner, coupled with the effectiveness of the Scan Ladder, suggests these tools could play a crucial role in advancing digital dental techniques. This is particularly relevant for complex full-arch edentulous scans, showing that the accuracy of intraoral scanners can approach that of traditional lab scanners when the appropriate tools are employed. This presents clinicians with a viable and efficient alternative for creating digital dental implants.

However, this study acknowledges that without enhancements such as the Scan Ladder, traditional intraoral scanners face challenges in reliably capturing complete arches in edentulous patients. This underscores the need for continued innovation and the integration of new technologies to address these limitations effectively. While these results are promising, they are derived from a pilot study with a limited sample size and specific scanner configurations. To confirm the full potential and benefits of novel tools like the Scan Ladder and the Elite Intraoral Photogrammetry scanner, further research is necessary. Future studies should involve larger, more diverse clinical settings with a prospective design to provide a comprehensive understanding of these technologies’ applicability in everyday clinical practice.

## Figures and Tables

**Table 1:** The digital scanners used in this study.

Name	Manufacturer	Technology	STL Export	PLY/OBJ Colour Export	Photogrammetry
Primescan	Dentsply-Sirona, York, PA, USA	Structured light –Confocal microscopy with Smart Pixel sensor.	YES	YES (Indirect)	NO
i900	Medit, Seongbuk-gu, Seoul, Korea	Structured light-Active Speed 3D Video™	YES	YES	NO
Elite	Shining 3D Tech Co., Hangzhou, China	Intraoral Photogrammetry	YES	YES	YES—Intraoral

**Table 2:** Mean trueness and standard deviation of each scanner and each scan type in comparison to the master scan from the Ineos X5 in order of ascending mean deviation and significance compared to the Ineos X5 results.

Name	Mean (μm)	Std. Deviation (μm)	*p* Value
Primescan	80.6	18.3	<000.1
Primescan (novel method 1—indirect)	37.6	11.4	<000.1
Primescan (novel method 2—TiDirect)	22.2	10.6	<000.1
Medit i900	86.9	17.2	<000.1
Medit i900 (novel method 1—indirect)	38.6	12.5	<000.1
Medit i900 (novel method 2—TiDirect)	22.7	10.3	<000.1
Elite Intraoral Photogrammetry	18.7	9.5	<000.1

**Table 3:** Tukey homogenous subsets of compared means (subset for alpha = 0.05).

Name	1	2	3	4
Elite Intraoral Photogrammetry	18.7			
Primescan (novel method 2—direct)	22.2			
Medit i900 (novel method 2—direct)	22.7			
Primescan (novel method 1—indirect)		37.6		
Medit i900 (Novel Method 1—Indirect)			38.6	
Primescan				80.6
Medit				86.9
p value (Sig)	1.000	1.000	1.000	0.052

## Data Availability

The original contributions presented in the study are included in the article, further inquiries can be directed to the corresponding author.
